# Randomized controlled phase IIa clinical trial of safety, pharmacokinetics and pharmacodynamics of tenofovir and tenofovir plus levonorgestrel releasing intravaginal rings used by women in Kenya

**DOI:** 10.3389/frph.2023.1118030

**Published:** 2023-06-13

**Authors:** Nelly R. Mugo, Victor Mudhune, Renee Heffron, Katherine K. Thomas, Eleanor McLellan-Lemal, Betty Njoroge, Sue Peacock, Siobhán M. O’Connor, Beatrice Nyagol, Eunice Ouma, Renee Ridzon, Jeffrey Wiener, Nina Isoherranen, David W. Erikson, Louise A. Ouattara, Nazita Yousefieh, Terry A. Jacot, Richard E. Haaland, Susan A. Morrison, Harald S. Haugen, Andrea R. Thurman, Shannon A. Allen, Jared M. Baeten, Taraz Samandari, Gustavo F. Doncel

**Affiliations:** ^1^ International Clinical Research Center, Department of Global Health, University of Washington, Seattle, WA, United States; ^2^ Center for Clinical Research, Kenya Medical Research Institute (KEMRI), Nairobi, Kenya; ^3^ HIV Research Division, Centre for Global Health Research, Kenya Medical Research Institute (KEMRI), Kisumu, Kenya; ^4^ Department Epidemiology, University of Washington, Seattle, WA, United States; ^5^ Division of HIV Prevention, National Center for HIV, Viral Hepatitis, STD, and TB Prevention, Centers for Disease Control and Prevention, Atlanta, GA, United States; ^6^ Department of Pharmaceutics, University of Washington, Seattle, WA, United States; ^7^ Endocrine Technologies Core, Oregon National Primate Research Center, Oregon Health & Science University, Beaverton, OR, United States; ^8^ CONRAD, Eastern Virginia Medical School, Norfolk, VA, United States; ^9^ Office of HIV and AIDS, Bureau for Global Health, United States Agency for International Development, Washington, DC, United States; ^10^ Department of Medicine, University of Washington, Seattle, WA, United States; ^11^ Division of HIV Prevention, National Center for HIV, Viral Hepatitis, STD, and TB Prevention, Centers for Disease Control and Prevention (United States), Kisumu, Kenya

**Keywords:** intravaginal ring, multipurpose technology, tenofovir, levonorgestrel, HIV, HSV-2, Africa

## Abstract

**Introduction:**

Globally, many young women face the overlapping burden of HIV infection and unintended pregnancy. Protection against both may benefit from safe and effective multipurpose prevention technologies.

**Methods:**

Healthy women ages 18–34 years, not pregnant, seronegative for HIV and hepatitis B surface antigen, not using hormonal contraception, and at low risk for HIV were randomized 2:2:1 to continuous use of a tenofovir/levonorgestrel (TFV/LNG), TFV, or placebo intravaginal ring (IVR). In addition to assessing genital and systemic safety, we determined TFV concentrations in plasma and cervicovaginal fluid (CVF) and LNG levels in serum using tandem liquid chromatography-mass spectrometry. We further evaluated TFV pharmacodynamics (PD) through *ex vivo* CVF activity against both human immunodeficiency virus (HIV)-1 and herpes simplex virus (HSV)-2, and LNG PD using cervical mucus quality markers and serum progesterone for ovulation inhibition.

**Results:**

Among 312 women screened, 27 were randomized to use one of the following IVRs: TFV/LNG (*n* = 11); TFV-only (*n* = 11); or placebo (*n* = 5). Most screening failures were due to vaginal infections. The median days of IVR use was 68 [interquartile range (IQR), 36–90]. Adverse events (AEs) were distributed similarly among the three arms. There were two non-product related AEs graded >2. No visible genital lesions were observed. Steady state geometric mean amount (ssGMA) of vaginal TFV was comparable in the TFV/LNG and TFV IVR groups, 43,988 ng/swab (95% CI, 31,232, 61,954) and 30337 ng/swab (95% CI, 18,152, 50,702), respectively. Plasma TFV steady state geometric mean concentration (ssGMC) was <10 ng/ml for both TFV IVRs. *In vitro*, CVF anti-HIV-1 activity showed increased HIV inhibition over baseline following TFV-eluting IVR use, from a median of 7.1% to 84.4% in TFV/LNG, 15.0% to 89.5% in TFV-only, and −27.1% to −20.1% in placebo participants. Similarly, anti-HSV-2 activity in CVF increased >50 fold after use of TFV-containing IVRs. LNG serum ssGMC was 241 pg/ml (95% CI 185, 314) with rapid rise after TFV/LNG IVR insertion and decline 24-hours post-removal (586 pg/ml [95% CI 473, 726] and 87 pg/ml [95% CI 64, 119], respectively).

**Conclusion:**

TFV/LNG and TFV-only IVRs were safe and well tolerated among Kenyan women. Pharmacokinetics and markers of protection against HIV-1, HSV-2, and unintended pregnancy suggest the potential for clinical efficacy of the multipurpose TFV/LNG IVR.

**Clinical Trial Registration:**

NCT03762382 [https://clinicaltrials.gov/ct2/show/NCT03762382]

## 1. Introduction

Women accounted for 49% of the estimated 1.5 million new HIV infections in 2021 a majority of whom reside in sub-Saharan Africa, where girls and women represent 63% of new HIV infections (1). Globally, 64% of the estimated 0.5 billion persons infected with herpes simplex virus (HSV-2) are women (2–4). Human immunodeficiency virus-1 (HIV-1) and HSV-2 have a synergistic relationship, with a two-fold increased risk for HIV among HSV-2 infected persons (5). In sub-Saharan African countries with endemic HIV, adolescent girls and young women aged 15–24 years account for 24% of incident HIV infections although they comprise only 10% of the population (5). In the United States and other Western countries, an estimated 19% of incident HIV infections occur among women, with 85% of these attributed to heterosexual transmission (6). Concurrently, pregnancy related complications remain the leading cause of death among girls aged 15–19 years in low-income countries, with approximately 10 million unintended pregnancies each year in this age group (7). Young women face triple epidemics of HIV, unintended pregnancy and HSV-2 infection.

Multipurpose prevention technologies (MPTs) aim to simultaneously meet sexual and reproductive health needs, including prevention of unintended pregnancies, HIV infection, and other sexually transmitted infections (STIs) with a single product. Therefore, MPTs have the potential to provide significant reproductive health benefits to women globally (8). Market research has demonstrated that women in sub-Saharan Africa would prefer MPTs conferring protections against both HIV and unintended pregnancies instead of separate methods (9). Long-acting, female-controlled MPT interventions have the potential to overcome barriers that limit use of existing preexposure prophylaxis (PrEP) products, such as adherence, stigma, lack of privacy for storing products, and perception of HIV risk (10). Findings from a recent systematic review of intravaginal ring (IVR) acceptability and preference among women in low- and middle-income countries reported that women expressed a preference for accessible, long-acting products that can be used covertly without partner knowledge and with few side effects (11).

CONRAD, a non-profit biomedical research and development organization, developed two 90-day controlled-release IVRs containing tenofovir (TFV) alone (TFV-only) or TFV/levonorgestrel (LNG), which were both similar in appearance to the contraceptive NuvaRing® (12, 13). The CONRAD A13–128 trial evaluated both IVRs for safety, pharmacokinetics (PK), pharmacodynamics (PD) and drug release with 15-day use among healthy, sexually-active, low-risk women in the United States and the Dominican Republic (14). Both IVRs were found to be safe, with vaginal TVF concentrations above 100,000 ng/ml, higher than the 489 ng/swab estimated threshold for HIV prevention (15). LNG plasma concentrations among TFV/LNG IVR users were above the 240 pg/ml threshold for systemic LNG contraceptive efficacy and cervical mucus Insler score with abnormal sperm penetration (14). Building on these results, we assessed the TFV-releasing IVRs with and without LNG during up to 90-day use for safety, PK, and PD in a study among women in Western Kenya.

## 2. Materials and methods

CONRAD Protocol B17–144 was a single site, phase IIa randomized, partially blinded, placebo-controlled clinical trial conducted at the Jaramogi Oginga Odinga Referral Hospital, Center for Global Health Research clinic, Kenya Medical Research Center (KEMRI), Kisumu, Kenya from December 14, 2018, to August 20, 2019. Institutional ethics review boards of KEMRI and the University of Washington reviewed and approved the study protocol. The protocol was registered in Clinicaltrials.gov (NCT03762382) and implemented in accordance with Good Participatory Practice guidelines, with engagement of a local community advisory board. Participant safety oversight was provided by a safety monitoring committee. Written informed consent was obtained from all participants prior to undertaking any study procedures.

Eligible women were aged 18–34 years; not pregnant; seronegative for HIV and hepatitis B surface antigen (HBsAg); ovulating (based on home use of an ovulation prediction kit) followed by confirmatory luteal phase serum progesterone (P4) ≥3.0 ng/ml; had a body mass index ≤30 kg/m^2^; scored ≤4 on a validated HIV risk scoring tool (predicted HIV incidence <3.95/100 person-years for women in sub-Saharan Africa) (16); and were not using or desiring to use PrEP and not planning to be pregnant during the study period. Prior to enrollment, women must have stopped using oral contraceptive pills for ≥2 months, injectable contraceptives for ≥4 months, or a contraceptive implant for ≥6 months. Prior use of contraceptive was assessed through self report, serum progesterone at screening visit and LNG detection in a blood sample collected prior to IVR insertion. Eligible women were provided with non-spermicidal condoms and copper intrauterine device (IUD) for contraception. Women who chose to use copper IUD had a two month wait period between IUD insertion and study IVR randomization and insertion. Women were ineligible if they had any pelvic abnormalities or were diagnosed with an STI. Screening of participants included testing for *Chlamydia trachomatis, Neisseria gonorrhoeae, Trichomonas vaginalis*, candidiasis (based on wet mount), bacterial vaginosis (BV) diagnosed using Nugent scoring or Amsel's criteria and syphilis (17). Women diagnosed with BV received treatment and were re-assessed for eligibility.

### 2.1. Study schedule and randomization

Study participants had up to 13 scheduled study visits arranged outside of days with menstruation. Participants were scheduled to use the IVR for 90-days or until August 20, 2019, to coincide with the expiry date of the IVRs. IVR insertion was scheduled in the follicular phase of the menstrual cycle and confirmed by measuring luteal phase serum progesterone prior to IVR insertion at visit three. Participants were randomized 2:2:1 to continuous use of one of the following IVRs: TFV/LNG; TFV-only; or placebo [containing starch instead of active pharmaceutical ingredient (API)]. The randomization scheme was generated using permuted block randomization to ensure balanced arm assignment over the accrual period. The study investigators and clinic staff were blinded to the randomization.

The randomized study IVR was inserted and removed by the study clinician. Participants were instructed to keep the IVR in place for continuous use for the duration of the study. Demonstration of self-insertion and removal was done using a 3-D demonstration model and participants practiced self-insertion and removal using a placebo IVR so they could re-insert the study IVR if it was accidentally or intentionally removed. Adverse events (AEs) were evaluated through clinical history at all visits. At baseline before IVR insertion, post-insertion, 24- hours post-removal and all scheduled study visits in between, the study clinician visually assessed for genital AEs through speculum pelvic exam. During these visits, samples were collected for the following biomedical measurements: TFV levels in cervicovaginal fluid (CVF) collected by vaginal swab during pelvic exam by the clinician; and plasma TFV, serum LNG and serum sex hormone binding globulin (SHBG) levels for PK analyses.

Additional vaginal swabs to characterize the vaginal microbiome, secreted soluble genital tract proteins, and activity against both HIV-1 and HSV-2 (hence forward referred to as anti-HIV and anti-HSV-2 activity) were collected pre-IVR insertion and at IVR removal. Cervical mucus quality assessment using Insler score and P4 levels were done to evaluate for ovulation during the first and third menstrual cycle and timed using urinary luteinizing hormone (LH) (18). Partner involvement and HIV testing for partners was encouraged but not required. Participants were asked to refrain from sexual activities 24 h prior to IVR insertion visit and 48 h prior to the ovulatory assessments.

### 2.2. Study product

CONRAD developed the two IVRs, which release TFV with or without LNG in a controlled and sustained manner for at least 90 days; pre-clinical product development and initial clinical evaluation have been previously reported (12, 13). The API TFV was supplied by Gilead Sciences, Inc. (USA) and the API LNG was acquired from Industriale Chimica s.r.l. (Italy). Particle Sciences (Bethlehem, PA, USA) manufactured under good manufacturing practice (GMP) conditions and shipped clinical study products (IVRs) to the clinical site packaged in individual re-sealable foil pouches, ready to use. The rings were stored at room temperature (15°–30°C) since they did not require cold chain storage. Each study participant received an IVR containing either 1.15 g of TFV plus 6.0 mg of LNG (estimated daily release doses of 8–10 mg of TFV and 20 µg of LNG), 1.41 g TFV (estimated daily release dose of 8–10 mg of TFV), or a non-eluting placebo IVR. The TFV IVR consisted of a single segment of polyurethane tubing filled with a white TFV-containing paste. The TFV/LNG IVR appearance was similar to that of the TFV IVR except it contained a 2 cm-long solid hydrophobic polyurethane reservoir segment loaded with 6 mg LNG, capped by 2 mm-wide hydrophobic polyurethane spacers welded to the TFV segment. The placebo IVR had the same dimensions and configuration as the TFV/LNG IVR in which the TFV API is replaced by modified starch (that is non-eluting from the reservoir) to provide a similar white filled tube appearance and the short segment consists of solid polyurethane without LNG.

### 2.3. Safety outcomes

Grade 2 or higher genital and systemic treatment emergent AEs were primary study safety outcomes, including cervicovaginal ulcerations, abrasions, edema, or findings as assessed by naked eye visualization of the cervicovaginal epithelium, including at IVR removal. AEs were also defined by abnormal safety laboratory measurements. AEs were graded and assessed for relationship with use of study product and/or procedures by the study physician. A safety monitoring committee met every two weeks to review AEs. Each adverse event was graded for severity using the July 2017 update of the Division of AIDS (DAIDS) table (https://rsc.niaid.nih.gov/sites/default/files/daidsgradingcorrectedv21.pdf).

### 2.4. TFV and LNG pharmacokinetic assessment

TFV concentrations were quantified from plasma samples collected at IVR insertion and each visit until 24 h post-IVR removal. A Dacron swab was used to collect genital fluid from the lateral vaginal wall to quantify amount of TFV in genital fluid at baseline before ring insertion and at every visit following IVR insertion. TFV concentrations in plasma and amount on vaginal swabs were determined *via* protein precipitation followed by tandem mass spectrometry (LC-MS/MS) analysis as previously described (19, 20). The lower limit of quantification (LLOQ) of TFV for this study was 10 ng/ml for plasma and 1 ng/swab for vaginal swabs. Assay-specific results with concentrations below the lower limits of quantification were imputed as 1/2*LLOQ. Serum LNG concentrations were measured by the Endocrine Technologies Core at the Oregon National Primate Research Center (ETC ONPRC) with a Shimadzu Nexera-LCMS-8050 liquid chromatography-tandem triple quadrupole mass spectrometry (LC-MS/MS) platform (Shimadzu Scientific, Kyoto, Japan) using a previously published method (21). Briefly, LNG was extracted from samples using supported liquid extraction and LNG concentrations were then determined by LC-MS/MS across two assays. The assay range was 20 pg/ml–10 ng/ml; intra- and inter-assay coefficients of variation were <10%. Free LNG index was computed as the ratio of LNG nmol/L to SHBG nmol/L after converting LNG to nmol/L per molar mass of 312.446 g.

### 2.5. Pharmacodynamic (PD) assessments

#### 2.5.1. Levonorgestrel PD assessment

We modeled the potential contraceptive efficacy of LNG by assessing several surrogates, including ovulation during IVR use, defined as a serum P4 ≥3.0 ng/ml at months 1, 2, and 3; at IVR removal; and 24 h after IVR removal. Study participants started checking for LH surge on day 10 of the menstrual cycle (after IVR insertion) at home using ovulation prediction kits (OPK) and presented within 12–24 h post LH surge. To evaluate local micro-dose LNG effects, at least two examiners assessed the cervical mucus Insler score on a scale of 0–3 for each factor (Spinnbarkeit, volume, viscosity, cellularity and ferning) with a combined score of 10 or more indicating normal, ovulatory, mid cycle mucus receptive to sperm penetration (18).

#### 2.5.2. Anti-HIV-1 & anti-HSV-2 PD assessment in cervicovaginal fluid

##### 2.5.2.1. Activity against HIV

For CVF activity against HIV-1 (PD), CVF was collected from the lateral vaginal wall using Dacron swabs, which were then frozen until analysis. Testing was performed at the CONRAD Intramural Laboratory at Eastern Virginia Medical School (Norfolk, VA, USA) using the TZM-bl cell line (ATCC: The Global Bioresource Center | ATCC)) as previously reported (22). Briefly, TZM-bl cells were plated and CVF (1:5 or 1:10 final dilution in DMEM/10% FBS/1% penicillin/streptomycin) was applied to the appropriate wells. For toxicity testing, 100 µl of medium with or without CVF were added to each well for 48 h. For antiviral evaluation, Bright-Glo Luciferase Assay System (Promega, Madison, WI, USA) was used following the manufacturer's instructions. Briefly, 100 µl of medium, with or without CVF, containing HIV-1BaL (5 × 10^3^ TCID50) were added to each well. After 48 h, the cells were lysed with 100 µl of Glo Lysis buffer. Lysates (50 µl) were then transferred to a 96 well black microtiter plate and 50 µl of Bright-Glo assay reagent added before luminescence was measured in a BioTeK microplate reader. The average percent inhibition of HIV-1BaL growth in wells exposed to CVF was determined based on deviations from HIV-1 only control. Within the same participant, antiviral activity was further assessed comparing HIV-1 infection in the presence of CVF collected at IVR removal to infection in the presence of baseline CVF.

##### 2.5.2.2. Activity against HSV-2

Using Starplex™ Scientific Starswab II™, CVF was collected from the lateral vaginal wall, frozen and stored at −80°C until processing. Thawed swabs were placed in 300 µl of HEC1A media for 5–10 min, then placed in SpinX insert (MIDSCI, M850003) and centrifuged at 370 g force for 5 min at 4°C to remove all secretions from the swab. To assess the activity of the swab eluent against HSV-2, HEC1A cells (ATCC: The Global Bioresource Center | ATCC) were plated at 200,000/well in a 48 well plate containing McCoy's 5A medium with penicillin/streptomycin. The following day 70 µl of the swab extract were added to the well for a total of 6 h and in the last hour, each well was infected with 200 PFU HSV-2 in 30 µl of media. The treatment/inoculum was removed and 200 µl of fresh media were added. Real-time polymerase chain reaction (PCR) was done on Day 5 using the supernatant to detect HSV-2 DNA and compared to untreated control.

#### 2.5.3. Soluble immune mediators in cervicovaginal secretions

Soluble markers were eluted from CVF collected with Dacron swabs (14). Cytokines interleukin (IL)-1β, IL-6, IL-8, IL-10, tumor necrosis factor-alpha (TNFα), granulocyte macrophage colony-stimulating factor (GM-CSF), regulated upon activation, normal T cell expressed and secreted (RANTES), interferon-*γ*-inducible protein 10 (IP-10), macrophage inflammatory protein 1a (MIP-1α), and IL-1 receptor agonist (IL-1 RA) were measured in swab eluents using Luminex technology (25 µl of sample) (Millipore, Billerica, MA, USA). Secretory leukocyte protease inhibitor (SLPI) (R&D Systems, Inc., Minneapolis, MN, USA) and human *β* defensins 1, 2, and 3 (Alpha Diagnostics, San Antonio, TX, USA) were quantified by commercial enzyme-linked immunosorbent assay and read using a Varioskan LUX multimode microplate reader (ThermoFisher Scientific, Waltham, MA, USA). Soluble markers were reported as concentration per swab.

#### 2.5.4. Residual drug assessments and estimated in vivo drug release rates

Details on analysis of the LNG IVR segment have been previously described (12–14). Used IVRs containing TFV with or without LNG were stored in individual sealed foil packages at −80°C until shipped on dry ice to Lubrizol Health Services (Bethlehem, PA, USA) for evaluation of residual drug (14). IVRs containing LNG segments were cut at the joint between the LNG segment end cap and the end of the sealed TFV segment to isolate the LNG segment.

Analysis of LNG by LC-MS/MS was conducted similar to methods previously described (21, 23). IVR release rates were estimated by subtracting the recovered API concentration result from the average control API recovery and dividing by the number of days of reported use. Serum LNG concentrations were measured by the Endocrine Technologies Core at the Oregon National Primate Research Center (ETC ONPRC) with a Shimadzu Nexera-LCMS-8050 liquid chromatography-tandem triple quadrupole mass spectrometry (LC-MS/MS) platform (Shimadzu Scientific, Kyoto, Japan) using a previously published method (21). The assay range was 20 pg/ml–10 ng/ml; intra- and inter-assay coefficient of variation were <10%. The IVR release rates were estimated by subtracting the recovered API concentration from the reference standard and dividing by the number of days of reported use. In an exploratory descriptive analysis (with a small sample size per group) we examined potential effects of BV-associated microbiota on TFV released and estimated release rates.

#### 2.5.5. Placebo IVR assessment

CONRAD Intramural laboratories assessed placebo IVRs for visual appearance as well as glycerin content to determine duration of use. At the time the IVR is inserted in the vagina, glycerin, an excipient in the TFV paste contained within the ring, is released in a time-dependent manner until most of its content is exhausted. Residual glycerin content, therefore, may be used as a marker indicating lack of use or low adherence (23).

#### 2.5.6. SHBG assessment

Plasma samples for SHBG assessment were collected at IVR insertion and each visit until 24 h post-IVR removal. SHBG levels were measured by the ETC ONPRC using a Roche cobas e411 automatic immunoassay (Roche Diagnostics, Indianapolis, IN, USA). The assay range for SHBG is 0.033–19 µg/ml; intra- and inter-assay coefficient of variation (*n* = 2 assays) were <2.8%.

#### 2.5.7. Assessment of vaginal microbiota

A lateral vaginal wall swab was collected, and a Gram stain performed to assess Nugent score prior to IVR insertion and at IVR removal visits (17). Absolute abundance of bacteria per swab was determined by quantitative PCR of the 16S region to determine the microbial composition of the female genital tract. The vaginal microbiota assessment was done at the Seattle Children's Hospital laboratories (Seattle, Washington, USA), as described in a separate publication (24).

### 2.6. Demographic, behavioral, and other participant characteristics

Demographic data and perceptions of sexual partner attitudes, as well as behavioral data on sexual behaviors and IVR acceptability were collected *via* audio computer-assisted personal interview. IVR adherence and tolerability data were collected *via* electronic case report forms.

### 2.7. Sample size and statistical analyses

The participant sample size was planned to be 50 based on feasibility, similarly sized phase I studies, and study timelines rather than statistical criteria.

### 2.8. Statistical analysis

Safety was evaluated by clinical review of descriptive statistics of AEs by randomization group. For the evaluation of PK endpoints, plasma TFV, vaginal swab CVF, and serum LNG were used to calculate the following, planned PK parameters: maximum concentration (C_max_), concentration steady state (C_SS_), percent of steady state achieved 24 h after IVR insertion, concentration 24 h after the IVR removal visit; and the area under the curve (ln/linear trapezoidal method). Geometric means and 95% confidence intervals (CI) for PK parameters were calculated assuming a log-normal distribution. To evaluate the effect, if any, of the TFV/LNG product combination, transformed TFV concentrations were compared between participants randomized to the TFV-only IVR vs. the TFV/LNG IVR. TFV concentrations were compared using mixed log linear models, with treatment group as fixed effect and time (visit) as a repeated measure. Since the trial was not powered to find statistically significant group differences in primary or secondary endpoints, inferences based on statistical significance (or lack thereof) are made cautiously. Changes in soluble markers and anti-HSV-2 and anti-HIV-1 activity over time using IVR was assessed statistically by comparing paired measurements from pre-IVR insertion visit and IVR removal visit, using the Wilcoxon Signed Rank test.

## 3. Results

**Figure 1:**
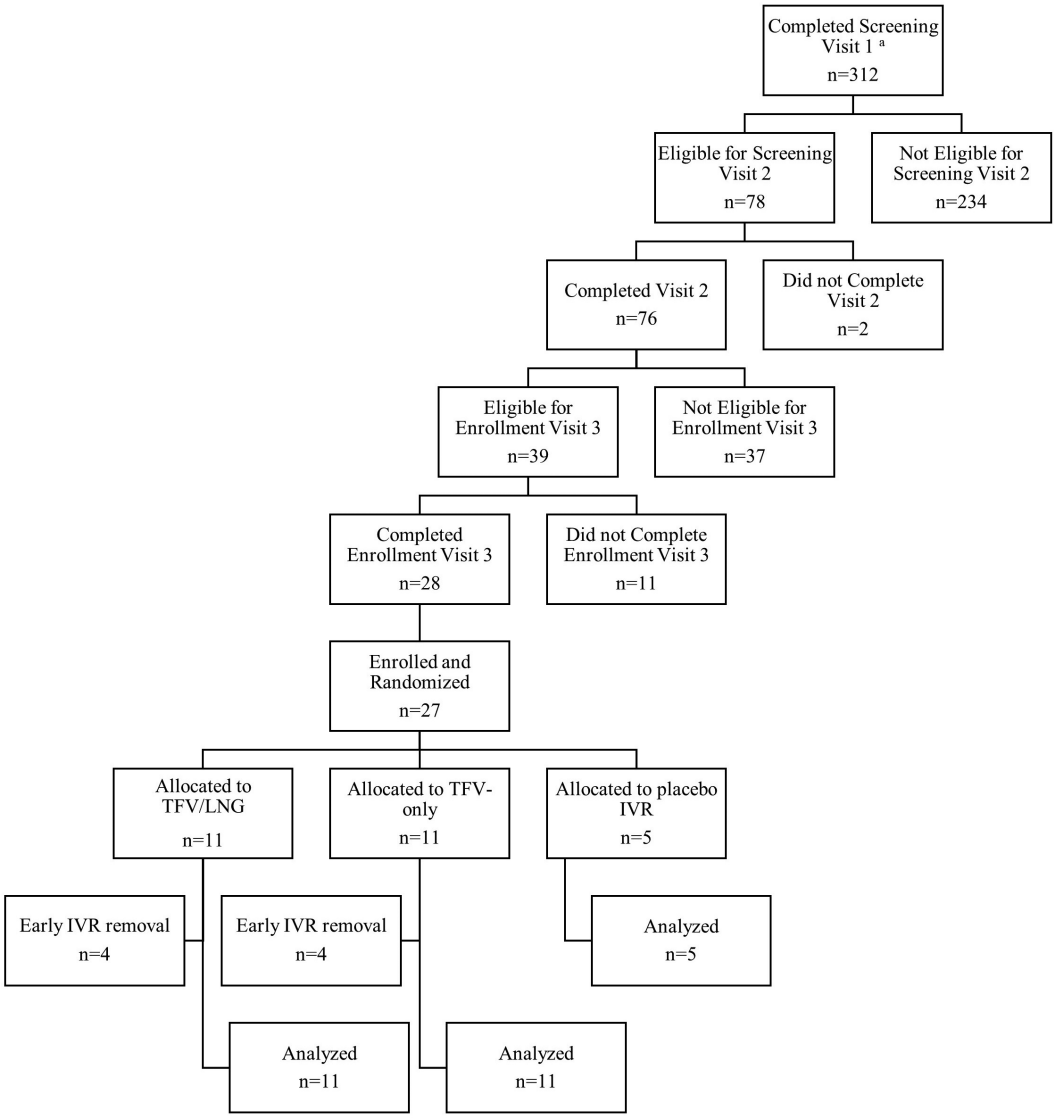
CONRAD Protocol B17-144 screening and enrollment flow chart, Kisumu, Kenya, 2019. ^a^Provided written informed consent including data and sample collection and storage for study screening and enrollment.

The first study IVR was inserted on January 31, 2019, and the study concluded in September 2019. As summarized in Figure 1, 312 women completed clinic screening visits and 27 eligible women were randomized to IVR insertion. The most common reasons for screening failures were bacterial vaginosis (BV) (32.6%) and positive STI test results (HIV, syphilis, *Neisseria gonorrhoeae, Chlamydia trachomatis* or hepatitis B virus) (27.4%). Less prevalent reasons included Grade 2+ laboratory abnormalities and inability to confirm ovulatory cycles (Supplementary Table S1). Eleven women were randomized to TFV/LNG, 11 to TFV-only and five to a placebo IVR use. The mean age of enrolled women was 24 years (SD 4.7), 24 (88.9%) had some secondary school education and 13 (48%) had been previously pregnant (Table 1).

**Table 1:** Participant demographic and sexual health characteristics by treatment group, Kisumu, Kenya, 2019.

Characteristics	Treatment group			
	TFV/LNG IVR (*n* = 11)	TFV-only IVR (*n* = 11)	Placebo IVR (*n* = 5)	Overall (*n* = 27)
Age, mean (SD)^a^	22.09 (2.88)	25.91 (5.74)	23.80 (4.76)	23.96 (4.74)
BMI, mean (SD)^b^	21.83 (3.78)	23.38 (3.10)	23.03 (3.45)	22.69 (3.40)
Menstrual cycle length^a^^,^^c^, mean (SD)	31.14 (4.15)	29.29 (4.20)	31.00 (4.54)	30.42 (4.12)
Previously pregnant^a^	4 (36.4%)	6 (54.5%)	3 (60.0%)	13 (48.1%)
Education^a^				
At least some primary school	0 (0.0%)	1 (9.1%)	2 (40.0%)	3 (11.1%)
At least some secondary school	9 (81.8%)	8 (72.7%)	2 (40.0%)	19 (70.4%)
Completed college/university	2 (18.2%)	2 (18.2%)	1 (20.0%)	5 (18.5%)
Marital status				
Single	8 (72.7%)	7 (63.6%)	4 (80.0%)	19 (70.4%)
Married	3 (27.3%)	3 (27.3%)	1 (20.0%)	7 (25.9%)
Divorced/separated	0 (0.0%)	1 (9.1%)	0 (0.0%)	1 (3.7%)
Sexually active in past 3 months (*n* = 20)^a^				
Yes	8 (88.9%)	7 (100.0%)	3 (75.0%)	18 (90.0%)
No	1 (11.1%)	0 (0.0%)	1 (25.0%)	2 (10.0%)
Contraceptive use in past 6 months^a^^,^^d^				
None	1 (9.1%)	2 (18.2%)	1 (20.0%)	4 (14.8%)
Oral contraceptives	2 (18.2%)	2 (18.2%)	0 (0.0%)	4 (14.8%)
Male condom	9 (81.8%)	8 (72.7%)	4 (80.0%)	21 (77.8%)
Intrauterine device	1 (9.1%)	1 (9.1%)	0 (0.0%)	2 (7.4%)
Nugent score				
Nugent score at IVR insertion^b^, median (IQR)	0.00 (0.00–5.00)	0.00 (0.00–0.00)	0.00 (0.00–7.00)	0.00 (0.00–5.00)
Positive for BV^e^ at screening^a^	0 (0.0%)	0 (0.0%)	0 (0.0%)	0 (0.0%)
Positive for BV^e^ at IVR insertion^b^	1 (9.1%)	1 (9.1%)	2 (40.0%)	4 (14.8%)

TFV, tenofovir; LNG, levonorgestrel; IVR, intravaginal ring; SD, standard deviation; BMI, body mass index; IQR, interquartile range; BV, bacterial vaginosis.

^a^ Data collected at screening visit where participants were allowed to skip the question.

^b^ Data collected just preceding (at time of) IVR insertion (baseline, Visit 3).

^c^ Menstrual cycle length estimated from dates recorded on a Screening Menstrual Bleeding Electronic Case Report Form. The average of 2 cycles was taken for women who provided 3 dates, otherwise cycle length is computed from 2 dates. Eight women had just one menstrual period start date recorded and therefore are not included in computation of cycle length.

^d^ A participant could report more than 1 type of contraception.

^e^ Nugent score ≥7 was interpreted as positive for BV. Nugent score assessment occurred at both screening visit and IVR insertion visit (baseline, Visit 3).

### 3.1. Duration of IVR use

The median duration of IVR use was 68 days [interquartile range (IQR) 36–90]; 46 days (IQR 21–89) among women randomized to the TFV/LNG, 90 days (IQR 40–91) for the TFV-only, and 68 days (IQR 67–90) for the placebo group. No study participant was lost to follow up and only one scheduled study visit was missed. Six (22%) women had unplanned early IVR removal. Among the TFV/LNG IVR group, four women had early IVR removal at day 21, 34, 36 and 46 of IVR use for the following reasons: one due to menorrhagia, two due to symptomatic BV, and one due to vulvovaginitis and recurrent IVR dislodgement. In the TFV IVR group, two women had early removal due to pregnancy confirmed at day 16 and 63 of IVR use; there were no early removals in the placebo IVR group.

### 3.2. Safety

A total of 110 AEs occurred in 26 women across the intervention and control arms, 58 (53%) grade 1 and 50 (45%) grade 2, one grade 3 and one grade 4 but only 7 (6%) were determined to be related to the study product. The grade 4 and grade 3 AEs were determined to be unrelated to the study intervention. AEs were similarly distributed among the three groups of IVR users (Table 2 and Supplementary Table S2).

**Table 2:** Adverse events^*^ by treatment group, Kisumu, Kenya, 2019.

	TFV/LNG IVR *n* (%)	TFV-only IVR *n* (%)	Placebo IVR *n* (%)	Overall *n* (%)
Adverse events (AEs)^a^				
Total AEs (any grade)	47 (42.7%)	47 (42.7%)	16 (14.5%)	110 (100.0%)
Serious AEs	0 (0%)	1 (100.0%)	0 (0%)	1 (100.0%)
Participants reporting at least one AE				
AE (any grade)	11 (100.0%)	10 (90.9%)	5 (100.0%)	26 (96.3%)
Severity of AE^b^				
Grade 1: Mild	0 (0%)	1 (10%)	1 (20%)	2 (8%)
Grade 2: Moderate	11 (100%)	8 (80%)	4 (80%)	23 (88%)
Grade 3: Severe	0 (0%)	0 (0%)	0 (0%)	0 (0%)
Grade 4: Potentially life-threatening	0 (0%)	1 (10%)	0 (0%)	1 (4%)
Grade 5: Death	0 (0%)	0 (0%)	0 (0%)	0 (0%)
Relationship of AE to IVR use^c^				
Any related AE	6 (55%)	1 (10%)	0 (0%)	7 (27%)
No related AE	5 (45%)	9 (90%)	5 (100%)	19 (73%)

TFV, tenofovir; LNG, levonorgestrel; IVR, intravaginal ring; AE, adverse event(s).

^*^ July 2017 update of the Division of AIDS (DAIDS) table (https://rsc.niaid.nih.gov/sites/default/files/daidsgradingcorrectedv21.pdf).

^a^ Events reported. More than one event may have been reported per participant. Percentages given are among total events reported and represent a row percent.

^b^ Participants reporting more than one AE were counted only once using the highest severity of AE reported.

^c^ Participants reporting more than one AE were counted only once using the closest relationship to IVR use reported (i.e., “related” or “not related”).

Among the 27 enrolled women, the most reported AEs were BV in 12 (44.4%) women with 12 events, headache in 10 (37%) with 13 events, and upper respiratory tract infections (URTIs) in seven (25.9%) women with seven events, with similar distribution across study groups (Supplementary Table S2). Among the diverse etiologies of grade 2 AEs, the most common was URTI in six (22%) participants, BV in five (19%), reduced estimated glomerular filtration rate (eGFR) in five (19%), vulvovaginitis in four (15%) and headache in four (15%) (Supplementary Table S2). Additional AE data is summarized in Table 2 and Supplementary Table S2.

### 3.2.1. Systemic adverse events

There were five reported grade 2 AEs, with reduced eGFR compared to baseline in five (18.5%) women, one of which followed acute malaria and another a complete abortion; three women were in the TFV/LNG and two in TFV-only group. Decreases in eGFR from baseline values were limited to a range of 10.0% to <30.0% change, and all eGFR remained >90 ml/min/1.73 m^2^, within normal parameters and assessed to not have clinical significance. One participant in the TFV only group, who experienced a complete abortion after the IVR removal visit also had grade 3 AE with reduced sodium reported at the final visit.

### 3.2.2. Genitourinary (GU) tract adverse events

Menstrual cycle changes with IVR use were observed in all three groups. Most AEs associated with menstrual changes were in the TFV/LNG group, in which five women reported intermenstrual bleeding, one had prolonged bleeding that led to IVR removal, and one had heavy menstrual bleeding. In the TFV group, one woman reported a grade 1 menstrual change AE with increased menstrual bleeding; none of the placebo IVR users had changes reported as AEs. Only one of these AEs was grade 2 and presented with prolonged light bleeding for 20 days, was assessed as product-related and led to product discontinuation. The other three GU AEs that led to product discontinuation were grade 2 BV and vulvovaginitis. Among 11 women in the TFV-only group, there were 17 GU AEs, with five grade 2AEs, and one grade 1 AE related to menstrual disorder. Among the placebo group, there were three GU AEs, two due to BV (grade 1 & 2) and one to genital pruritus (grade 1). There were no product discontinuations related to GU AEs in the TFV-only and placebo groups.

BV was the most common GU AE, with seven grade 1 and five grade 2 diagnosed in 12 women after IVR insertion—four (36.4%) women in the TFV/LNG, six (54.5%) in the TFV-only and two (40%) in the placebo IVR group. There was one visible small nodular vaginal lesion in the genitalia noted after IVR insertion in the TFV/LNG group, which was not product use related.

### 3.3. Acceptability and adherence

Women expressed concerns about using the IVR prior to use, but most concerns diminished with use. At the study screening visit, 73% of participants expressed some concern about the IVR but after IVR use only one woman in the TFV/LNG group expressed physical discomfort once or twice with IVR use and no one had difficulty with removal. Two women in the TFV-only IVR group reported removing the IVR for less than 2 h and had no difficulty with re-insertion. One woman in the TFV/LNG group had IVR displacement in the vagina which she easily repositioned. There were no IVR expulsions. Three women in the TFV/LNG group expressed concern with bleeding irregularities. At exit, 60% stated they would use an IVR for HIV prevention alone, all would use an IVR for both pregnancy and HIV prevention and all would recommend the IVR to their community.

Residual glycerin content, assessed only in the placebo IVRs, was high in the IVRs of two women, suggesting low adherence to use. All women in the placebo group stated they did not remove the IVR, did not feel any discomfort and did not feel it inside the vagina. Residual TFV and LNG assessed in women using TFV containing rings was consistent with IVR use and demonstrated steady depletion with each additional day of reported use (Supplementary Figures S1A, S1B).

### 3.4. Tenofovir and levonorgestrel pharmacokinetics assessment

#### 3.4.1. Tenofovir in cervicovaginal fluid

**Figure 2:**
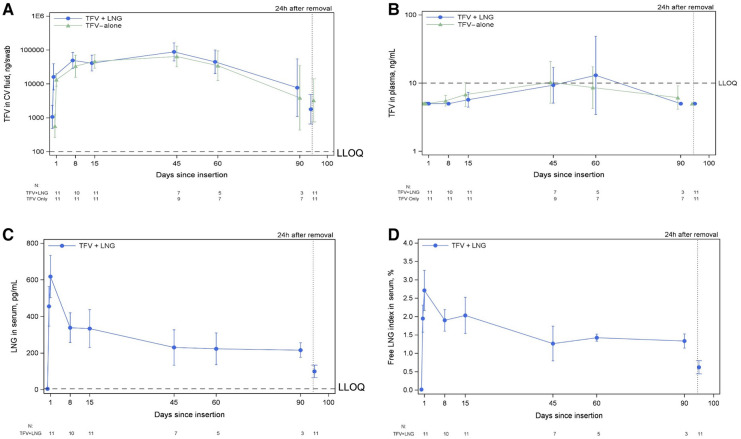
Tenofovir (TFV) levels among TFV/LNG and TFV-only intravaginal ring (IVR) users, and LNG levels among TFV/LNG IVR users, Kisumu, Kenya, 2019. 2A: TFV concentration in cervicovaginal (CV) fluid from IVR insertion through 24 hours (24h) after IVR removal; 2B: Plasma TFV concentration from IVR insertion through 24h after IVR removal; 2C: Serum LNG concentration from IVR insertion through 24h after IVR removal; 2D: Serum free LNG index from IVR insertion through 24h after IVR removal. LLOQ, lower limit of quantification.

In both TFV-containing IVR treatment groups, there was a rapid increase in TFV levels in vaginal fluid following insertion (Figure 2A). At 6 h post-IVR insertion, median vaginal fluid TFV was 1,300 ng/swab (IQR 638–3,520) in the TFV/LNG group and 837 ng/swab (IQR 419–1,290) in the TFV-only IVR group. At the 24-hour sampling, the geometric mean amount (GMA) of TFV was 16,141 ng/swab (95% CI 6,549, 39,784) in the TFV/LNG group and 13,208 ng/swab (95% CI 8,532, 20,446) in the TFV-only IVR group, and at steady state, 43,988 ng/swab (95% CI 31,232, 61,954) in the TFV/LNG group and 30,337 ng/swab (95% CI 18,152, 50,702) in the TFV-only IVR group. Time to maximum (T_max_) TFV GMA in cervicovaginal swabs was similar in the two treatment groups. For the TFV/LNG IVR group, T_max_ was 26.1 days (95% CI 16.1, 36.1), and for the TFV-only IVR group, T_max_ was 34.4 days (95% CI 17.6, 51.1). There was immediate decline in TFV vaginal amounts within 24 h of IVR removal, with GMA of 1,789 ng/swab (95% CI 645, 4,958) in the TFV/LNG group and 3,261 ng/swab (95% CI 745, 14,276) in the TFV-only IVR group. TFV GMA at 24 h post-insertion, during steady state, and at 24 h post-removal, as well as T_max_ or C_max_, were similar in both treatment groups (Figure 2A).

#### 3.4.2. Daily tenofovir release rates from IVRs

**Figure 3:**
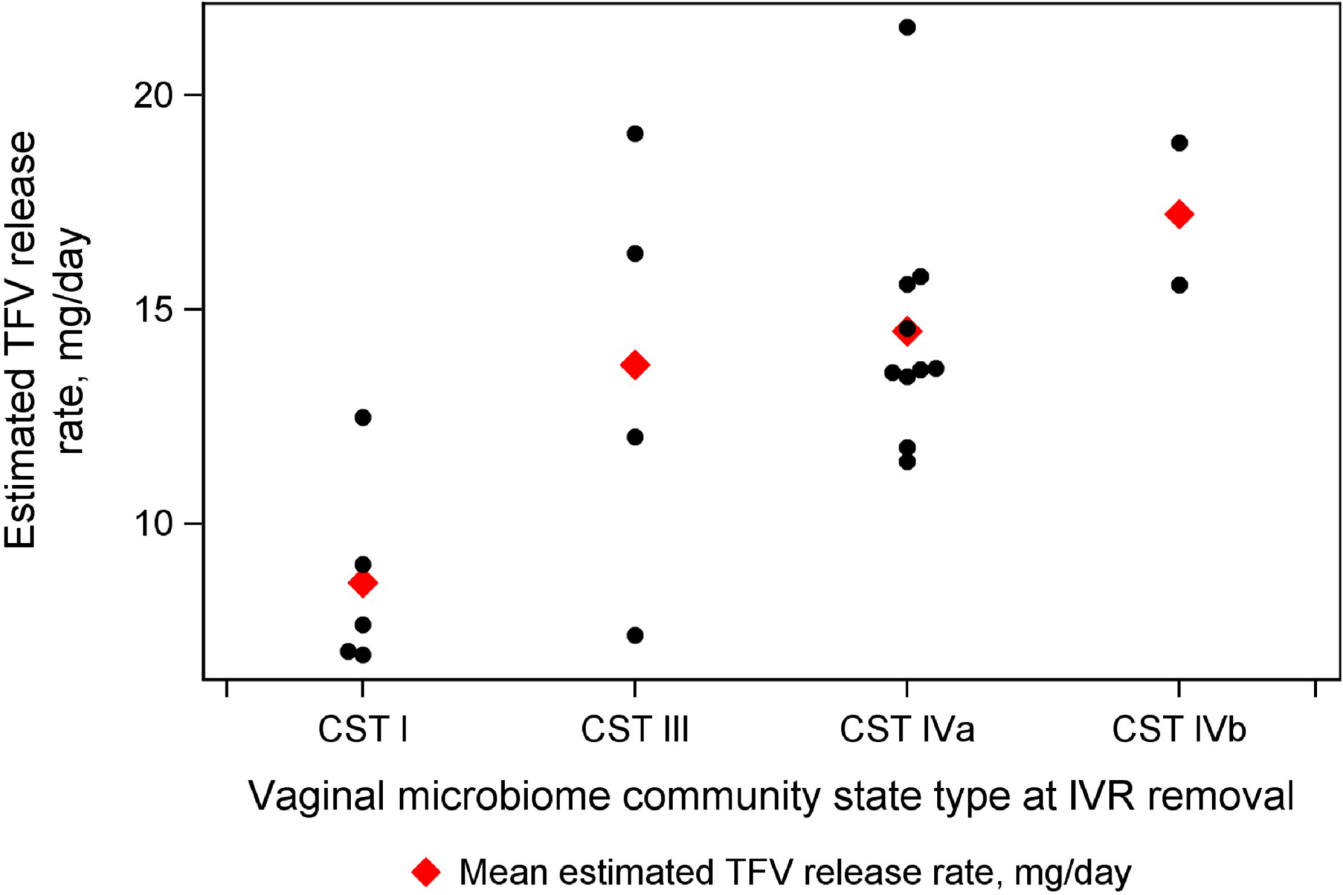
Estimated tenofovir (TFV) release rate, by vaginal microbiome community state type (CST) at intravaginal ring (IVR) removal visit. Kisumu, Kenya 2019.

Among the TFV/LNG and the TFV-only IVR groups, the estimated TFV release rate was similar at 12.3 mg/day (SD 4.2) and 14.0 mg/day (SD 3.8), respectively. The potential effect of BV-associated microbiota on TFV release was assessed on a smaller sample size per treatment group (24). The estimated daily release rate was 8.6 mg (SD 2.3) among women with *Lactobacillis crispatus*-dominant community state type (CST I), 13.7 mg (SD 5.1) among *Lactobacillus iners*-dominant community state type (CST III), and 14.5 (SD 2.9) among non-*Lactobacillus*-dominant state types (CST IV). Tenofovir release increased with increased bacterial diversity and BV-associated bacteria (Figure 3).

#### 3.4.3. Tenofovir in plasma

At 6 h prior to IVR insertion, 24 h post-insertion and throughout IVR use (with one exception at day 60), the steady state plasma concentration of TFV remained below the level of quantification (BLQ) (<10 ng/ml) for both TFV-containing IVR treatment groups), (Figure 2B).

#### 3.4.4. Levonorgestrel in serum

Mean serum LNG levels were BLQ (<7 pg/ml) prior to TFV/LNG IVR insertion with T_max_ of one day and exceeding 400 pg/ml within 6 h. LNG concentrations (GMC) were 586 pg/ml (95% CI 473, 726) 24 h after insertion, 241 pg/ml (95% CI 185, 314) during steady state, and 87 pg/ml (95% CI 64, 119) at 24 h after IVR removal (Figure 2C). Geometric mean serum free LNG index was 2.6% (95% CI 2.1%, 3.2%) at 24 h after insertion, 1.5% (95% CI 1.2%, 1.9%) during steady state, and 0.6% (95% CI 0.4%, 0.8%) at 24 h after IVR removal (Figure 2D).

#### 3.4.5. Daily levonorgestrel release rates from IVRs

Among the TFV/LNG IVR group, the estimated LNG release rate was 25.2 µg/day (SD 6.4).

### 3.5. Pharmacodynamics of tenofovir in cervicovaginal fluid

**Figure 4:**
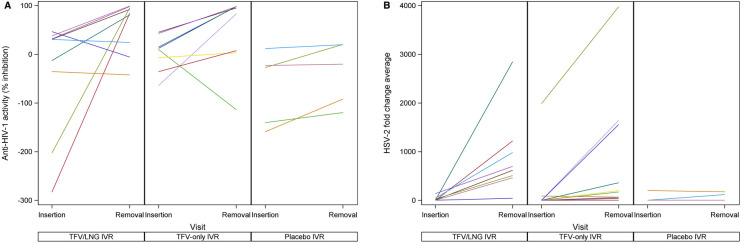
Cervicovaginal fluid (CVF) in-vitro human immunodeficiency virus, type 1 (HIV-1) and herpes simplex virus, type 2 (HSV-2) inhibition with tenofovir (TFV)/levonorgestrel (LNG), TFV-only, and placebo intravaginal ring (IVR) study groups. 4A: CVF in-vitro HIV-1 inhibition^a^; 4B: CVF in-vitro HSV-2 inhibition^a^. ^a^Note, the y axes in figures 4A and 4B start at different cut off points.

Activity against HIV-1 in CVF demonstrated a median of 7.1% inhibition at baseline, increasing markedly to a median of 84.4% at IVR removal (*p* = 0.05) for the TFV/LNG group. In the TFV-only IVR group median activity against HIV-1 also increased markedly from 15.0% inhibition at baseline to 89.5% at IVR removal (*p* = 0.15) (Table 3). In the placebo IVR group, the median activity against HIV-1 was similar at baseline and end of IVR use, −27.1% and −20.1% inhibition, respectively (Table 3). The TFV-only IVR group includes a few outliers displaying low/no inhibitory activity similar to that of the placebo group (Figure 4A). Among the TFV IVR groups, six of 17 women for whom CVF could be evaluated at IVR removal exhibited low HIV inhibition. Four of these had used the IVR for 90 days, had high estimated average TFV release rates and, at IVR removal, low levels of intravaginal TFV, low residual IVR TFV content and BV-associated microbiota (CST IV). The other two women had the IVR removed at 20–34 days and, at IVR removal, had high CVF TFV concentrations and IVR TFV content and showed CST III or IV (one each) microbiota. Regarding CVF activity against HSV-2, the median log_10_ fold-change reduction in HSV-2 levels at baseline and IVR removal was 8.8 and 563.7, respectively, in the TFV/LNG group (*p* = 0.008), and 1.8 vs. 185.9, respectively, in the TFV-only group (*p* = 0.006). In the placebo group, there was little change in HSV−2 levels from a baseline log_10_ fold−change median of 102.2 (IQR 2.4–711.5) to 119.3 (IQR 2.3–177.3) at IVR removal (Table 3 and Figure 4B), indicating no increase in CVF anti-HSV-2 activity due to placebo IVR use.

**Table 3:** HIV-1 and herpes simplex, type 2 (HSV-2) inhibition activities in cervicovaginal fluid, by randomized treatment group and visit type, Kisumu, Kenya, 2019.

	Treatment group		
	TFV/LNG IVR (*n* = 11)	TFV-only IVR (*n* = 11)	Placebo IVR (*n* = 5)
Activity against HIV-1, % inhibition			
Pre-IVR insertion (*n*)	11	11	5
Median (IQR)	7.10 (−35.4–31.70)	15.00 (−6.90–37.30)	−27.10 (−140–−22.9)
At IVR removal (*n*^a^)	9	8	5
Median (IQR)	84.40 (24.50–95.30)	89.45 (5.45–98.10)	−20.10 (−91.80–19.90)
*p*-value change from pre-IVR insertion^b^	0.05	0.15	0.06
Activity against HSV−2, fold change^c^^,^^d^			
Pre-IVR insertion, (*n*^c^)	9	10	4
Median (IQR)	8.77 (2.49–44.14)	1.80 (1.35–5.82)	102.2 (2.36–711.5)
At IVR removal (*n*^c^)	10	10	3
Median (IQR)	563.7 (43.22–983.3)	185.9 (61.15–1,558)	119.3 (2.33–177.3)
*p*-value change from pre-IVR insertion^b^	0.0008	0.006	1.000

HIV-1, human immunodeficiency virus, type 1; HSV-2, herpes simplex virus, type 2; TFV, tenofovir; LNG, levonorgestrel; IVR, intravaginal ring; IQR, interquartile range.

^a^ Five participants samples were contaminated and are not included in the results.

^b^ *p*-values for comparison of differences from IVR pre-insertion to end of treatment using Wilcoxon signed-rank test for paired values.

^c^ Results of 4 specimens were not conclusive due to contamination or cytotoxicity and are not included in the results.

^d^ Inhibition fold change = 1/fold change between log10 quantity by PCR in control (medium–only) sample, and log10 quantity by PCR in tested sample where CVF is included.

### 3.6. Pharmacodynamics of levonorgestrel

#### 3.6.1. P4 and sex hormone binding globulin (SHBG)

All study participants had a luteal phase serum progesterone (P4) ≥3.0 ng/ml prior to IVR insertion. Serum P4 measurements with IVR use relative to pre-IVR insertion were consistently lower in the TFV/LNG group and were not substantially different in the TFV-only and placebo groups (Table 4a). At day 20–25 of the first menstrual cycle, only four (36.4%) women in the TFV/LNG group had P4 ≥3.0 ng/ml, indicating ovulatory cycles, while nine (81.8%) in the TFV-only and five (100.0%) in the placebo group showed values above 3.0 ng/ml. This trend continued to be seen at day 20–25 of the second menstrual cycle and at the IVR removal visit. One woman in the TFV-only group had detectable LNG pre-IVR, and at 6- and 24-hours post IVR insertion. SHBG levels in serum declined by 68% from baseline levels to IVR removal in the TFV/LNG group but remained similar to baseline levels in the TFV-only and placebo groups through IVR use.

**Table 4a:** Surrogates of contraceptive efficacy: Serum progesterone (P4) levels at intravaginal ring (IVR) removal, randomized by treatment group and visit, Kisumu, Kenya, 2019.

P4 (ng/ml)	Treatment group		
	TFV/LNG IVR (*n* = 11)	TFV-only IVR (*n* = 11)	Placebo IVR (*n* = 5)
Visit 6: day 20–25 of 1st menstrual cycle			
Participants with P4 assessment	11	11	5
≥3	4 (36.4%)	9 (81.8%)	5 (100.0%)
<3	7 (63.6%)	2 (18.2%)	0 (0.0%)
Visit 7: day 20–25 of 2nd menstrual cycle^a^			
Participants with P4 assessment	7	9	5
≥3	3 (42.9%)	8 (88.9%)	4 (80.0%)
<3	4 (57.1%)	1 (11.1%)	1 (20.0%)
Day 90 end of treatment (EOT) visit, pre-IVR removal^b^			
Participants with P4 assessment	3	6	2
≥3	1 (33.3%)	3 (50.0%)	2 (100.0%)
<3	2 (66.7%)	3 (50.0%)	0 (0.0%)

P4, progesterone; IVR, intravaginal ring; TFV, tenofovir; LNG, levonorgestrel; EOT, end of treatment.

^a^ One participant is missing P4 at Visit 7; five participants discontinued IVR use prior to Visit 7.

^b^ One participant is missing P4 at Visit 9; 15 participants discontinued IVR use prior to 90 days.

#### 3.6.2. Cervical mucus assessment

The overall mean length of the menstrual cycle was 30 days (SD 4.1) across the three study groups. The mean length was 31 days (SD 4.1) in the TFV/LNG group, 29 days (SD 4.2) in TFV-only and 30 days (SD 4.5) in the placebo group. At day 14 of the first menstrual cycle after IVR insertion, six (60.0%) women in the TFV/LNG group, three (27.3%) in the TFV IVR group and one (20.0%) in the placebo group had an Insler cervical mucus score <7, reflecting poor cervical mucus (17). Similar findings were observed around day 14 (ovulatory) of the second cycle (Table 4b). The median cervical mucus score during the first menstrual cycle was 6 (IQR 5–8) for the TFV/LNG group, 9 (IQR 6–11) for the TFV-only group and 9 (IQR 9–10) for the placebo group.

**Table 4b:** Cervical mucus score (Insler Score, 0–15), simplified slide test, Kisumu, Kenya, 2019.

	Treatment group		
	TFV/LNG IVR (*n* = 11)	TFV-only IVR (*n* = 11)	Placebo IVR (*n* = 5)
Visit 5: Day 14 of 1st menstrual cycle^a^			
Observations	10	11	5
<7	6 (60.0%)	3 (27.3%)	1 (20.0%)
7–10	3 (30.0%)	5 (45.5%)	4 (80.0%)
Good (>10)	1 (10.0%)	3 (27.3%)	0 (0.0%)
Median (IQR)	6.0 (5.0–8.0)	9.0 (6.0–11.0)	9.0 (9.0–10.0)
Visit 8: Day 14 of 3rd menstrual cycle^b^^,^^c^			
Observations	5	7	4
<7	2 (40.0%)	1 (14.3%)	2 (50.0%)
7–10	3 (60.0%)	6 (85.7%)	1 (25.0%)
Good (>10)	0 (0.0%)	0 (0.0%)	1 (25.0%)
Median (IQR)	7.0 (6.0–8.0)	9.0 (7.0–10.0)	6.5 (5.5–10.0)

TFV, tenofovir; LNG, levonorgestrel; IVR, intravaginal ring; IQR, interquartile range.

^a^ One woman did not attend Visit 5; percentages are based on 26 participants.

^b^ Eleven women did not attend Visit 8; percentages are based on 16 participants.

^c^ Visit 8 for this table includes 12 women attending Visit 8, which was prior to end of treatment (EOT) IVR removal and 4 who attended Visit 9 (EOT IVR removal) at the time of Visit 8 visit window.

### 3.7. Soluble immune markers

Evaluation of 11 soluble immune mediators in CVF demonstrated an increase of five mediators in the TFV/LNG and five in the TFV-only group between pre- and post-insertion compared to three in the placebo group, although most of these changes were not statistically significant. The TFV-only group showed an increase in median IL-1a from 159 to 462 pg/ml (*p* < 0.001) and reduction in median secretory leucocyte protease inhibitor SLPI from 430,355 to 71598 pg/ml (*p* = 0.03) (Supplementary Table S3).

## 4. Discussion

In this study, IVRs releasing TFV only and TFV/LNG used continuously for up to 90 days (median duration 68 days) were safe and well tolerated by young, sexually active Kenyan women assessed to be at low risk for HIV infection. These findings are consistent with those from two trials conducted in the USA and the Dominican Republic using the same IVRs (14, 25). Median vaginal steady state TFV amounts over 1,000 ng/swab correlate well with the estimated threshold for HIV prevention (15, 26, 27). Furthermore, CVF from participants using TFV-based IVRs had evidence of *in-vitro* inhibitory activity against HIV-1 and HSV-2. Data from four of six TFV-containing ring users whose CVF did not exhibit HIV inhibition is consistent with depletion of TFV in the ring by the time the IVR was removed, as reported in the MTN-038 study (27). In the CAPRISA 004 effectiveness study, which evaluated event-driven pre- and post-coital TFV vaginal gel use, cervicovaginal aspirate TFV concentrations ≥1,000 ng/ml correlated with 76% HIV protection (28). In our study we assessed TFV in CVF per swab, as a more accurate way to present and compare cervicovaginal TFV levels. TFV concentration in cervicovaginal aspirates and swabs were found to correlate well in a prior study (14). Based on these findings, we propose that the CVF TFV concentrations observed in this study have potential to confer protection from HIV infection. High cervicovaginal TFV concentrations, both in fluid and tissues, and TFV-diphosphate, the active metabolite, in tissues, as well as high CVF viral inhibitory activity, were also reported in previous studies in populations of women from different parts of the United States and the Dominican Republic (21, 25, 27, 29).

Plasma TFV concentrations were BLQ throughout IVR use. This finding of low systemic absorption of tenofovir is similar across TFV-based microbicide studies (30–32) and previous TFV IVR studies (14, 15). The low plasma TFV concentration likely explains the lack of product-related systemic AEs with similar distribution between TFV-containing IVRs compared to placebo IVR users.

This study found no statistically significant changes in CVF soluble markers of immune activation and inflammation between IVR insertion and removal, except for a significant decrease in SLPI, an immune mediator previously shown to block HIV infection, and an increase in the inflammatory cytokine IL-1α (33, 34) in the TFV only group. The clinical significance of these two isolated findings, however, is unclear. In a phase I study of an unrelated tenofovir disoproxil fumarate (TDF) IVR, which raised safety concerns and stopped early due to several findings of grade 1 genital ulceration, the vaginal fluid of the TDF arm had multiple increased soluble inflammatory markers among users of the active TDF ring but not the placebo IVR (32). The mechanism of transport of TDF (a prodrug to TFV) differs from TFV (our study intervention product) and exposure of vaginal cells to equimolar concentrations of TDF compared to TFV has been shown to result in a ∼40-fold higher levels of the active metabolite, tenofovir diphosphate (35)*.* It is also possible that products of cleavage/degradation of the prodrug TDF delivered to a highly localized area of the mucosal might have contributed to its epithelial toxicity. The TFV rings tested in this study, collectively, have safety data from four studies assessing safety and PK of the rings used for up to 90 days, and in each of these studies, there was no evidence of vaginal ulcerations or vaginal inflammatory changes (14, 21, 36, 37).

Women diagnosed with BV at the screening visit in this study were not enrolled., During follow-up, however, BV was the most commonly identified AE and about 15% of women randomized to IVR use had asymptomatic BV. We observed a shift towards a healthier, less diverse vaginal microbiome with use of the TFV/LNG and placebo IVR and a slight shift towards more diverse community state with TFV-only IVR. These data have been reported separately (24). TFV degradation by BV-associated bacteria has been suggested as possible cause for the reported reduction in vaginal drug concentrations and TFV gel efficacy in the CAPRISA 004 study (26, 38). Contrary to this observation, in our study, TFV release rates were found to increase with vaginal bacterial diversity and BV-associated microbiota. The increased TFV release observed in the presence of BV or BV-associated microbiota, possibly linked to increased vaginal pH and its effect on TFV solubility, may have helped maintain TFV levels in the cervicovaginal compartment, at least for the median duration of use (68 days) and until the IVR content was exhausted (13). This unexpected change in IVR release kinetics may counter the postulated TFV luminal degradation and its deleterious effect on efficacy. Future follow up studies should further assess these changes and their impact on HIV prevention potential.

Changes in menstrual bleeding patterns were the only product-related AEs identified and were almost all in the TFV/LNG group. Among women using LNG hormonal contraceptive methods, irregular menstrual bleeding is common and may lead to contraceptive discontinuation (39). Changes in menstrual patterns with progestin only contraceptives however have not diminished their overall acceptability and share of the market. The TFV/LNG evaluated in this study delivers a microdose of LNG (∼20 µg/day), reducing anovulation and its associated menstrual changes and potentially increasing acceptability. The release rate of about 20 µg of LNG per day from the IVR is comparable to the LNG-intrauterine device, and lower than the two-rod Jadelle® contraceptive implant with an estimated *in vivo* LNG release of 100 µg per day (40). The lower LNG dose released by the TFV/LNG IVR was intended to reduce frequency of irregular bleeding while retaining contraceptive efficacy (41). Most of the TFV/LNG users in the CONRAD A15-138 study using similarly low dose of LNG in the IVR did not experience changes in menstrual bleeding (37). Serum LNG concentrations among TFV/LNG IVR users were above the estimated threshold of 200 pg/ml for effective contraception among systemic LNG users, suggesting contraceptive potential for this multipurpose prevention IVR (40, 42). This was further supported by mean serum LNG concentration of 400 pg/ml within 6-hour and T_max_ within 24-hours of use, meeting the standard minimum threshold of LNG serum concentration for contraception determined in early LNG contraceptive implant studies, from which concentrations of LNG above 210 pg/ml are held to infer contraceptive effectiveness (40). The threshold for serum LNG levels for contraceptive effectiveness with IVR LNG use, however, has not been determined. In our study, markers of fertility such as ovulation and cervical mucus quality suggest contraceptive potential for the TFV/LNG ring. Furthermore, there were no pregnancies in that group, while two pregnancies were registered among women using the TFV-only IVR.

In this study, steady state LNG GMC remained above the standard threshold of 200 pg/ml, with a quick drop to 87 pg/ml within 24 h of IVR removal, providing the basis for quick return to fertility. However, this rapid decline can also leave women unprotected if they delay insertion of a new IVR or re-insertion after self-removal. On the other hand, TFV-diphosphate in tissue remains high after removal for several days, endowing the IVR with a longer forgiveness for HIV protection (14).

LNG implants and intrauterine systems act to prevent pregnancy through suppression of ovulation, suppression of endometrial lining maturation and thickening of cervical mucus (43–45). In long term implant studies, while more than 50% of women resume ovulation and cycling, they still remain protected against pregnancy, presumably due to local effects on the female genital tract (45, 46). In this study, TFV/LNG IVR users predominantly had low cervical mucus Insler score, indicative of cervical mucus that is impenetrable by sperm. Furthermore, 57%–67% of these women had anovulatory cycles.

The small sample size is a major limitation of this study and did not allow us to characterize detailed changes in vaginal microbiota or immune soluble markers and their effect on TFV release rates. Other limitations include the need to limit duration of IVR use in some participants due to product expiration date, the fact that we did not take genital biopsies for PK or histology to avoid increasing the risk of acquiring genital infections, such as HIV, and the use of the glycerin-based adherence assay only in the placebo arm. The duration of ring use was different in the two arms of the study; however this cannot be attributed to AEs or other product related differences.

This is the first study conducted among women in Africa to evaluate two IVRs releasing TFV and TFV/LNG. Data showed the IVRs were acceptable, safe and well tolerated in this small sample of selected Kenyan women. High vaginal TFV and serum LNG concentrations for the median duration of use and consistent PK profile and surrogates of protection against HIV-1, HSV-2 and pregnancy suggest good potential for these vaginal rings as multipurpose prevention technologies, expanding choice and prevention tools among adolescent girls and women.

## Data Availability

The original contributions presented in the study are included in the article/supplementary materials, further inquiries can be directed to the corresponding author/s.
