# Supplementary material for: Randomized controlled phase IIa clinical trial of safety, pharmacokinetics and pharmacodynamics of tenofovir and tenofovir plus levonorgestrel releasing intravaginal rings used by women in Kenya
Source: Front Reprod Health. 2023 Jun 13;5:1118030. doi: 10.3389/frph.2023.1118030 (PMC10293630; doi:10.3389/frph.2023.1118030)
Supplement: Supplementary file 1 [file Datasheet1.pdf]

TABLE S1. Most common indications for ineligibility[a] (N=679), Kisumu, Kenya, 2019

Visit	Type of event	Number of events
**Pre-screen**		
	HIV risk score > 4	22
	Unwilling to use IVR	10
	STI (HIV, syphilis, TV, GC, CT)	8
	Relocating out of study area	8
	Menstrual cycle pattern	7
	Pregnancy	6
	Breastfeeding	5
**Screening Visit 1**		
	BV Nugent Score ≥ 7	92
	STI (HIV, syphilis, TV, GC, CT)	78
	Grade 2+ laboratory results	38
	Decline consent[b]	25
	Use of oral contraceptives	14
	Pregnancy	13
	Abnormal findings (lab, clinical, medical, social)	13
	BMI ≥ 30 kg/m^2	12
	Symptomatic BV	9
	Alcohol/drug use	8
	Not in good general health	6
	Vulvar/vaginal symptoms	5
**Screening Visit 2**		
	P4 < 3.0 ng/ml	32

HIV, human immunodeficiency virus; IVR, intravaginal ring; STI, sexually transmitted infection; TV, *Trichomonas vaginalis*; GC, *Neisseria gonorrhoeae*; CT, *Chlamydia trachomatis*; BV, bacterial vaginosis; P4, progesterone.

[a]A woman could experience multiple events in one visit.

[b]Provided written informed consent for pre-screening data collection.

TABLE S2. Most commonly occurring adverse events[a] (AEs) by treatment group, Kisumu, Kenya, 2019

Preferred Term[b]	Treatment Group (by Type of IVR)				
	TFV/LNG (n=11)	TFV-only (n=11)	Placebo (n=5)	Overall (n=27)	
				Events	Women
Bacterial vaginosis	4 (36.4%)	6 (54.5%)	2 (40.0%)	12	12 (44.4%)
Headache	4 (36.4%)	4 (36.4%)	2 (40.0%)	13	10 (37.0%)
Upper respiratory tract infection	3 (27.3%)	2 (18.2%)	2 (40.0%)	7	7 (25.9%)
Vulvovaginitis	4 (36.4%)	2 (18.2%)	0 (0.0%)	6	6 (22.2%)
Glomerular filtration rate decrease	3 (27.3%)	2 (18.2%)	0 (0.0%)	5	5 (18.5%)
Metrorrhagia	4 (36.4%)	1 (9.1%)	0 (0.0%)	7	5 (18.5%)
Back pain	2 (18.2%)	2 (18.2%)	0 (0.0%)	4	4 (14.8%)
Gastroenteritis	2 (18.2%)	1 (9.1%)	1 (20.0%)	4	4 (14.8%)
Malaria	1 (9.1%)	2 (18.2%)	1 (20.0%)	4	4 (14.8%)
Abdominal pain	0 (0.0%)	1 (9.1%)	2 (40.0%)	3	3 (11.1%)
Blood glucose increased	1 (9.1%)	1 (9.1%)	0 (0.0%)	2	2 (7.4%)
Blood sodium decreased	1 (9.1%)	1 (9.1%)	0 (0.0%)	2	2 (7.4%)
Dizziness	0 (0.0%)	1 (9.1%)	1 (20.0%)	2	2 (7.4%)
Dysuria	1 (9.1%)	1 (9.1%)	0 (0.0%)	2	2 (7.4%)
Hemoglobin decreased	0 (0.0%)	2 (18.2%)	0 (0.0%)	2	2 (7.4%)
Hyperglycemia	0 (0.0%)	1 (9.1%)	1 (20.0%)	2	2 (7.4%)
Low density lipoprotein increase	0 (0.0%)	1 (9.1%)	1 (20.0%)	2	2 (7.4%)
Pelvic pain	0 (0.0%)	2 (18.2%)	0 (0.0%)	2	2 (7.4%)
Urinary tract infection	1 (9.1%)	1 (9.1%)	0 (0.0%)	3	2 (7.4%)
Blood pressure diastolic increased	1 (9.1%)	0 (0.0%)	0 (0.0%)	2	1 (3.7%)

AE, adverse event(s); IVR, intravaginal ring; TFV, tenofovir; LNG, levonorgestrel.

[a]All other AEs occurred less than twice.

[b]MedDRA version 21.1.

TABLE S3. Inflammatory response at intravaginal ring (IVR) insertion and end of treatment pre-IVR removal, Kisumu, Kenya, 2019

Soluble marker (pg/mL) [a, b]	Pre-IVR insertion, median	Pre-IVR removal, median	p-value
**TFV/LNG IVR (n=11)**			
GM-CSF[c]	1.61	1.61	0.943
IL-1α	137.53	266.27	0.240
IL-6	10.08	28.61	0.765
IL-8	1909.00	4734.00	0.320
IL-1Ra	103700.0	256400.0	0.365
IL-10[d]	1.65	1.65	0.438
CCL3	17.25	46.67	0.577
CCL5	19.71	20.76	0.966
TNFα[e]	0.80	0.80	0.520
β-defensin 2[f]	81.26	162.80	0.641
CXCL10/IP-10 [g]	48.81	25.19	0.365
SLPI	132397.0	37369.00	0.413
**TFV-only IVR (n=11)**			
GM-CSF[c]	1.15	1.61	0.652
IL-1α	159.02	461.81	<0.001
IL-6	5.73	9.38	0.320
IL-8	1229.00	3012.00	0.083
IL-1Ra	388700.0	74170.00	0.320
IL-10[d]	1.65	1.65	0.188
CCL3	26.89	49.20	0.700
CCL5	12.14	5.64	0.413
TNFα[e]	0.56	0.56	0.375
β-defensin 2[f]	31.25	31.25	0.219
CXCL10/IP-10[g]	59.38	45.23	0.359
SLPI	430355.0	71598.00	0.032

Soluble marker (pg/mL) [a, b]	Pre-IVR insertion, median	Pre-IVR removal, median	p-value
**Placebo IVR (n=5)**			
GM-CSF [c]	1.84	0.64	0.313
IL-1α	400.14	377.25	0.625
IL-6	14.06	18.74	0.625
IL-8	1069.00	9420.00	0.125
IL-1Ra	255700.0	31450.00	0.125
IL-10[d]	1.65	1.65	0.500
CCL3	17.25	17.71	0.313
CCL5	18.63	2.93	0.125
TNF-α[e]	1.27	1.27	0.875
β-defensin[f]	149.40	31.25	0.375
CXCL10/IP-10[g]	24.91	34.09	0.313
SLPI	79060.00	94661.00	0.813

IVR, intravaginal ring; TFV, tenofovir; LNG, levonorgestrel; GM-CSF, granulocyte-macrophage colony-stimulating factor; IL, interleukin; IL-1Ra, IL-1 receptor antagonist; CCL3, chemokine (C-C motif) ligand 3 (macrophage inflammatory protein 1α); CCL5, chemokine (C-C motif) ligand 5; TNF-α, tumor necrosis factor-alpha; CXCL10, C-X-C motif chemokine ligand 10; IP-10, interferon gamma-induced protein 10; SLPI, secretory leukocyte protease inhibitor.

[a]A total protein content assay to normalize values was not done.

[b]All markers except IL-8, IL-1Ra, β-defensin 2, and SLPI contained extrapolated values that fall out of normal range. CCL3, CCL5, TNFα, β-defensin 2, CXCL10/IP-10 and IL-10, also have imputed values when measures fell out of normal range and could not be extrapolated. Markers with extrapolations or imputations accounting for more than 40% of reported values described in detail in other footnotes.

[c]87% of values fall below normal range and are extrapolated.

[d]74% of values fall below normal range and are extrapolated; 20% of values are imputed.

[e]82% of values fall below normal range and are extrapolated; 6% of values are imputed.

[f]48% of values are imputed.

[g]Lab unable to retrieve any usable value for 2 specimens.

[h]p-values computed using Wilcoxon signed-rank test for paired values.

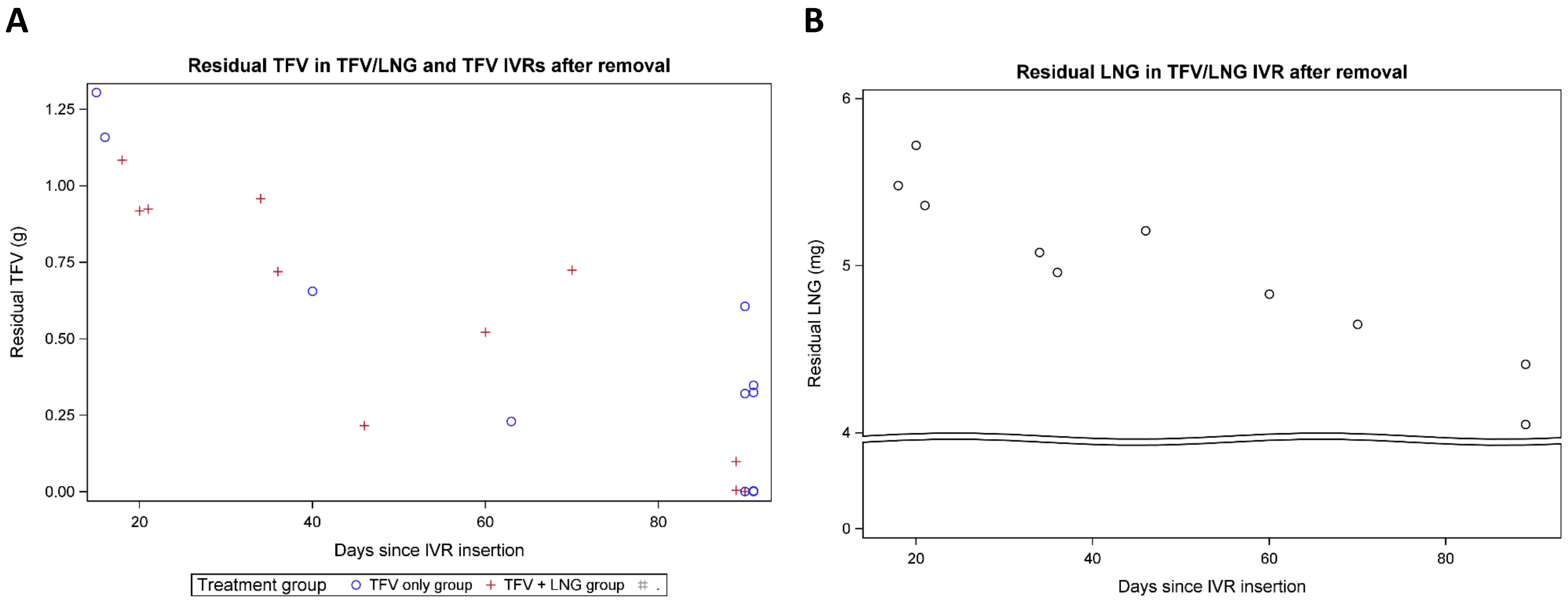

FIGURE S1

Residual tenofovir (TFV) and levonorgestrel (LNG) in TFV/LNG intravaginal rings (IVRs) and residual TFV in TFV-only IVRs after IVR removal, Kisumu, Kenya, 2019. S1A: Residual TFV in TFV/LNG and TFV-only IVRs after removal; S1B: Residual LNG in TFV/LNG IVRs after removal; y-axis break inserted between values 0.5-4 to improve graph readability.
